# Adaptive Single Photon Compressed Imaging Based on Constructing a Smart Threshold Matrix

**DOI:** 10.3390/s18103449

**Published:** 2018-10-14

**Authors:** Wentao Shangguan, Qiurong Yan, Hui Wang, Chenglong Yuan, Bing Li, Yuhao Wang

**Affiliations:** 1 School of Information Engineering, Nanchang University, Nanchang 330031, China; wentaoshangguan@outlook.com (W.S.); wanghuiyy35@163.com (H.W.); 13361646796@163.com (C.Y.); iamlibing_417816@163.com (B.L.); wangyuhao@ncu.edu.cn (Y.W.); 2 State Key Laboratory of Transient Optics and Photonics, Xi’an Institute of Optics and Precision Mechanics, Chinese Academy of Sciences, Xi’an 710119, China

**Keywords:** adaptive sensing, adaptive signal detection, compressed sensing, image sampling, measurement matrix, single-photon compressed imaging

## Abstract

We demonstrate a single-photon compressed imaging system based on single photon counting technology and compressed sensing theory. In order to cut down the measurement times and shorten the imaging time, a fast and efficient adaptive sampling method, suited for single-photon compressed imaging, is proposed. First, the pre-measured rough images are transformed into sparse bases as a priori information. Then a smart threshold matrix is designed by using large sparse coefficients of the rough image in sparse bases. The adaptive measurement matrix is obtained by modifying the original Gaussian random matrix with the specially designed threshold matrix. Building the adaptive measurement matrix requires only one level of sparse representation, which means that adaptive imaging can be achieved quickly with very little computation. The experimental results show that the reconstruction effect of the image measured using the adaptive measurement matrix is obviously superior than that of the Gaussian random matrix under different measurement times and different reconstruction algorithms.

## 1. Introduction

Single-photon imaging is a method of imaging in extremely low light levels by detecting and counting individual photons. It has been widely used in biomedical imaging, night vision, LIDAR, and astronomical spectroscopy [1,2,3,4]. In order to achieve high-sensitivity imaging, several spatially resolved detectors such as Intensified CCD (ICCD), Electron Multiplier CCD (EM-CCD), and Avalanche Photodiode (APD) array have been developed.

ICCDs and EM-CCDs operating in photon counting mode require very high frame rates and very low circuit noise, so a deep cooling device is always needed, which results in a very high cost. As a result of manufacturing difficulties and unstable performance, the resolution of APD arrays is still low [5,6,7]. An alternative method to obtain high resolution image is scanning imaging with a point detector such as Geiger-mode Avalanche Photodiode (APD) [8], photomultiplier tube (PMT) [9], and small-APD arrays detector. However, this method reduces the stability of the system and significantly increases imaging time due to low photon collection efficiency.

The single-pixel imaging scheme, based on Compressed Sensing (CS) theory, provides a new solution to the problems above. In that scheme, the target is imaged on a digital micromirror device (DMD), and focused on a single point detector after being modulated by the DMD. A 2D image can be reconstructed by a series of values of light intensity, detected by a single point detector, and the measurement matrix loaded into the DMD. On the basis of CS theory, the single-pixel imaging scheme could reduce the required measurements and shorten the imaging time. On the basis of single-pixel imaging technology, in 2012, Yu et al. proposed a single-photon compressed imaging scheme, in which the single point detector is replaced by a single-photon detector [10]. That scheme proved to have higher sensitivity for imaging than the tradition Single-pixel imaging scheme.

Whereas, when performing high-resolution imaging, single pixel imaging has some defects such as needing a lot of measurements and large amount of calculations. Thus, it needs a long time for image reconstruction. To solve these problems, some researchers obtain a priori information with a few previous measurements, on the basis of which they construct an adaptive measurement matrix to reduce the number of measurements [11]. They propose that the optimal way for choosing the measurement matrix is to select the measurement matrix that makes differential entropy decrease fastest. However, a large amount of computation is needed to calculate the optimal measurement matrix in this arithmetic. Averbuch et al. proposed an adaptive compressed sampling scheme that directly uses sparse base instead of a random measurement matrix for imaging to achieve a high-quality reconstruction of the image and greatly reduces reconstruction time [12]. Abmann and Byer present an approach for adaptive computational ghost imaging [13]. They obtain a rough image first and apply wavelet transform to that image. Then they find the space area where the coefficient of the image is larger then a threshold in wavelet basis. Afterwards, they image these areas with a higher resolution to reconstruct images with higher resolution. As it needs multiple levels of wavelet transform and imaging, the adaptive process becomes extremely complicated and often requires multiple adaptive measurements. In addition, in the space area where the wavelet coefficient is small, that adaptive computational ghost imaging system has a low imaging accuracy and large image reconstruction error.

In this study, we set up a single-photon compressed imaging system, based on which we present a new adaptive approach for the system. First, we transform the rough image obtained previously into a sparse basis as a priori information. Then, using some large sparse coefficient in the sparse basis, we set up a threshold matrix. Furthermore, the adaptive measurement matrix is built by combining the threshold matrix with original the Gaussian random matrix. In this process, only one level of sparse representation is needed to set up the adaptive measurement matrix. Additionally, there is no decline in the resolution of any space area, thus the entire imaging space area can be reconstructed with extremely high quality.

## 2. Principle and Realization of Single-Photon Compressed Imaging

**Figure 1:**
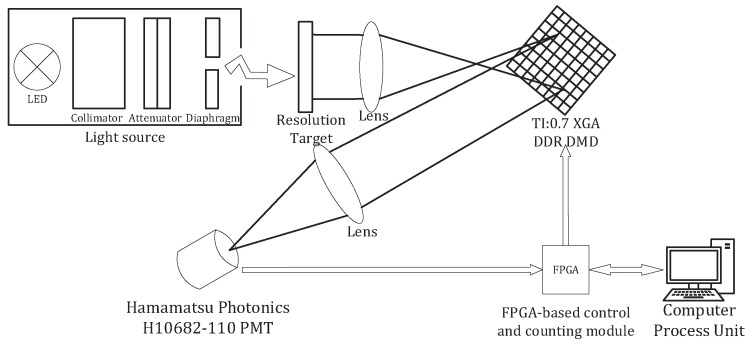
Schematic diagram of the single-photon compressed imaging system.

**Figure 2:**
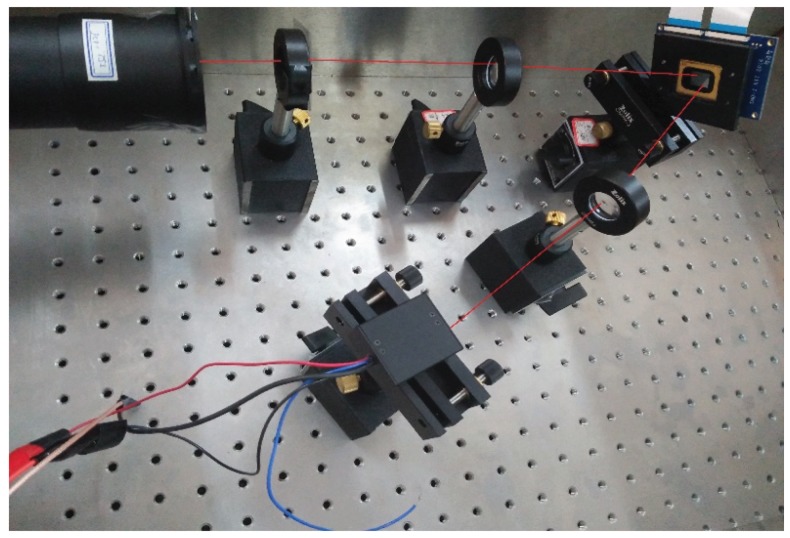
Photo of the light path of single-photon compressed imaging system.

The schematic diagram of the single-photon compressed imaging system is shown in Figure 1. Figure 2 shows the photo of the light path of our single-photon compressed imaging system. The light source consists of LED, collimator, attenuator, and diaphragm. After the light emitted by the LED passes through the collimator, the attenuator, and the aperture, it becomes a very weak parallel light whose intensity is on the single photon level. The target is imaged on a DMD by a lens. The DMD (TI:0.7 XGA DDR DMD) has 1024×768 micromirrors, the size of these is 13.68 μm × 13.68 μm. The DMD serves as the spatial light modulator by loading a random binary matrix into it. Each micromirror has two states of deflection. A deflection of +12 degrees and −12 degrees is represented by “0” and “1”, respectively. We placed a focusing lens in the +12 degree direction and collected the modulated light into a photon counting PMT (Hamamatsu Photonics H10682-110 PMT). As a point detector, the PMT can simultaneously collect the light intensity of multiple pixels on the imaging surface when performing one measurement. Therefore, the signal-to-noise ratio is very high, enabling imaging with higher detection sensitivity. A FPGA-based control and counting module is developed to load M random binary patterns into the DMD in order to achieve random modulation of the image on the DMD, and simultaneously record the number of photons collected by the detector after each modulation. The photon number corresponds to the intensity of the modulated light. After it is sent to the computer, the image is reconstructed based on CS theory.

In the single-photon compressed imaging system, the image focused on the DMD can be considered as an n-dimensional signal x. To modulate light and obtain the measurement $y_{i}$, which is the count value of a single photon pulse, DMD is loaded with each row of the binary measurement matrix $\Phi_{i}$. After all of the measurement matrix Φ is loaded, we get a series of measurement y (the dimension of x is usually smaller than y). The relationship between the signal x and measurement y can be described as:

(1) y=Φ·x

Essentially, based on CS theory, the reconstruction of the signal is a solution to the indefinite equation. Theoretically, the indefinite equation has an infinite set of solutions. Donoho, Emmanuel, Candes et al. formally proposed the CS theory in 2006 [14]. This theory indicates that the information obtained from a few linear measurements for sparse and compressible signals is quite sufficient to reconstruct the signal [15]. For the n-dimensional signal to be measured, it may not be a sparsity-based signal for itself. Whereas, the signal x can be sparsely represented in an N×N matrix $\Psi =[\begin{array}{cccc} \Psi_{1} & \Psi_{2} & \cdots & \Psi_{N} \end{array}]$. That is, the signal is compressible by transforming it into a sparse signal θ, then

(2) x=Ψ·θ

Then the measurement process Equation (1) could be rewritten as:

(3) y=Φ·x=Φ·Ψ·θ

Generally, let the M×N matrix $A^{\mathrm{CS}}=\Phi \cdot \Psi$. Usually, $A^{\mathrm{CS}}$ is called the sensing matrix. The reconstruction of sparse signal θ can be translated into the problem of minimizing the $l_{0}$ norm, then

(4) $$\min{∥\Psi \cdot \theta ∥}_{0}\;s.t.\;A^{\mathrm{CS}}\theta =\Phi \cdot \Psi \cdot \theta =y$$

Chen, Donoho, and Saunders proposed that if Φ and Ψ are not related, solving the problem of minimizing $l_{1}$ norm which is more simple can also produce the same solution [16], then

(5) $$\min{∥\Psi \cdot \theta ∥}_{1}\;s.t.\;A^{\mathrm{CS}}\theta =\Phi \cdot \Psi \cdot \theta =y$$

From the theory above, we can draw a conclusion that the sparsification of signal, the construction of measurement matrix, and the reconstruction algorithm are the cores of CS theory. The researchers have proposed several algorithms with high performances, like orthogonal matching pursuit (OMP) [17], regularized orthogonal matching pursuit (ROMP) [18], iterative hard thresholding (IHT) [19,20,21], and TV minimization by Augmented Lagrangian and ALternating direction ALgorithms (TVAL3) [22,23]. Involved in both the process of CS and the reconstruction of signal, therefore, the design of the measurement matrix affects the performance of the entire system. The measurement matrix can be divided into random measurement matrix [24,25] and adaptive measurement matrix. Common random measurement matrices include the Gaussian random matrix, the local Hadamard matrix, and the partial Fourier matrix. In this study, we present a fast and effective method to construct an adaptive measurement matrix to reduce the number of measurements and shorten the imaging time of the single-photon compressed imaging system.

## 3. The Construction of an Adaptive Measurement Matrix

First, in order to upload the matrix into the DMD, we should binarize the Gaussian random matrix $\Phi_{0}$. The effect of CS with the binarized measurement matrix Φ is comparable to that with the original Gaussian random matrix $\Phi_{0}$. The binarization algorithm is as follows:

(6) $$\Phi_{1}=\Phi_{0}+\left|{\Phi_{0}}_{\min}\right|$$

(7) $$\Phi =\left[\frac{\Phi_{1}}{{\Phi_{1}}_{\max}-{\Phi_{1}}_{\min}}\right]$$

where, [] is rounding-off method.

Select $K_{0}$ rows of the measurement matrix ${\hat{\Phi }}_{K_{0}\times N}$ from the binarized measurement matrix $\Phi_{M\times N}$ and roughly acquire the image with the selected matrix ${\hat{\Phi }}_{K_{0}\times N}$ in our single-photon compressed imaging system.

Thus, $K_{0}$ measured values $y_{0}$ are obtained as a priori information. Then, based on the measured values $y_{0}$, the coefficients θ of the image on the DCT basis Ψ could be calculated, which are also regarded as sparse signal. Suppose that is $\theta ={\left[\begin{array}{cccc} \theta_{1} & \theta_{2} & \cdots & \theta_{K_{0}} \end{array}\right]}^{{}^{\prime }}$. Generally, θ contains a limited number of large coefficients and it also contains some small coefficients that are close to 0. Then, according to many experiments, we create a threshold value $m=\frac{\theta_{\max}}{20}$ imposed on the sparse signal θ, where $\theta_{\max}$ is the largest element in $\theta ={\left[\begin{array}{cccc} \theta_{1} & \theta_{2} & \cdots & \theta_{K_{0}} \end{array}\right]}^{{}^{\prime }}$. In θ, filter out $K_{1}$ large coefficients larger than threshold value *m*, which are $\theta_{\gamma_{1}},\theta_{\gamma_{2}},\ldots ,\theta_{\gamma_{K_{1}}}$. Set the index of position of large coefficient to form an index vector $P=[\begin{array}{cccc} \gamma_{1} & \gamma_{2} & \cdots & \gamma_{K_{1}} \end{array}]$.

Then, we set a diagonal matrix Δ as a weight matrix. If n∈P, then let $\Delta_{n,n}=N_{max}$. Additionally, let the other diagonal elements in the matrix be set to $N_{min}$, In our experiment, $N_{max}$ is set to 1.8 and $N_{min}$ is set to 0.5, which can be described as:

(8) $$\Delta_{i,j}=\left\{\begin{array}{c} 0\;i\neq j\\ N_{\max}\;i=j\in P\\ N_{\min}\;i=j\notin P \end{array}\right.\;\left(i,j\in \left\{1,2,\ldots N\right\}\right)$$

Then, let threshold matrix $\tilde{\Delta }=\Psi \cdot \Delta \cdot \Psi^{-1}$. Among them, the settings of $N_{max}$ and $N_{min}$ all could affect the performance of CS.

If $\theta =\left[\begin{array}{cccccc} \theta_{1} & \theta_{2} & \theta_{3} & \theta_{4} & \cdots & \theta_{n} \end{array}\right]{}^{\prime }$ is the coefficient of the image in DCT basis, and we filter out large coefficient elements $\theta_{1},\theta_{2},\theta_{4}$, thus $P=[\begin{array}{ccc} 1 & 2 & 4 \end{array}]$. Then, the process for creating Δ is:

(9) $$\theta =\left[\begin{array}{c} \begin{array}{c} \theta_{1(big)}\\ \theta_{2(big)}\\ \theta_{3} \end{array}\\ \theta_{4(big)}\\ \vdots \\ \theta_{n} \end{array}\right]$$

(10) $$\Delta =\left[\begin{array}{cccccc} N_{\max} & & & & & \\ & N_{\max} & & & & \\ & & N_{\min} & & & \\ & & & N_{\max} & & \\ & & & & \ddots & \\ & & & & & N_{\min} \end{array}\right]$$

Let adaptive measurement matrix $\tilde{\Phi }=\Phi \cdot \tilde{\Delta }$, by connecting threshold matrix $\tilde{\Delta }$ and Gaussian random matrix Φ.

(11) $$\tilde{y}=\tilde{\Phi }\cdot \Psi \cdot \theta$$

So, with the adaptive measurement matrix, the mathematical model of the single-photon compressed imaging system can be expressed as:

(12) $$\tilde{y}=\Phi \cdot \Psi \cdot \Delta \cdot \Psi^{-1}\cdot \Psi \cdot \theta =\Phi \cdot \Psi \cdot \Delta \cdot \theta =\Phi \cdot \Psi \cdot \tilde{\theta }$$

When imaging with the adaptive measurement matrix, we could regard the imaging process as the observation for $\tilde{\theta }=\Delta \cdot \theta$. Through the modification of the threshold matrix Δ, the values of the small coefficients of the measurements $\tilde{\theta }$ are closer to zero, making the measurements $\tilde{\theta }$ more idealized. That is more suitable for reconstruction of the signal. Therefore, the reconstruction error using an adaptive measurement matrix $\tilde{\Phi }$ is much smaller than when using a Gaussian random matrix Φ, thus the performance of CS is better. Candes [26], Tao [24] et al. proposed that the measurement matrix needs to satisfy the Restricted Isometry Property (RIP) [25]. Gaussian random matrix Φ approximates the RIP [27,28,29], and is almost irrelevant to any sparse signal [30].

## 4. Experimental Results and Discussion

On the single-photon compressed imaging system shown in Figure 1, we loaded the Gaussian random matrix ${\hat{\Phi }}_{K_{0}\times N}$ for the first measurement to obtain a priori information of the image, based on which the adaptive measurement matrix is built by combining the threshold matrix with the original Gaussian random matrix. Finally, the adaptive measurement matrix is loaded onto the DMD through the FPGA-based control and counting module to perform the second measurement. According to many experiments, it is concluded that setting $N_{max}$ to 1.8 and setting $N_{min}$ to 0.5 can achieve better performance of CS. In our experimental condition, the sparse ratio of both measurement matrices is 0.2. The resolution of the image reconstructed is 64×64. Each acquisition takes 1 s. PMT receives the photons of the image of target on the entire DMD, and the mean of photon counting rate for each acquisition is 1801 cps. Accordingly, the light intensity of the image on the DMD is calculated to be 4.624 × 10${}^{-2}$ pW/cm${}^{2}$. The noise count of the system is 50 cps/s. Thus, according to the definition of noise equivalent power (NEP) [31], the light intensity of detection limit in the DMD is calculated to be 3.631 × 10${}^{-4}$ pW/cm${}^{2}$. In the following, we compare the performance of the Gaussian random matrix and the adaptive measurement matrix in terms of number of measurements, reconstruction algorithms, and noise immunity.

### 4.1. Effect of Measurement Times on Imaging Quality

**Figure 3:**
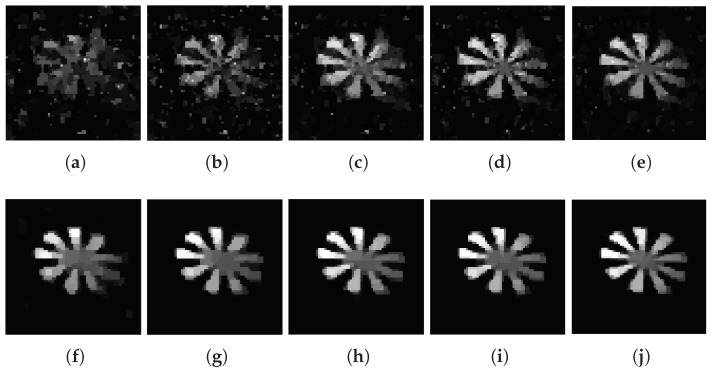
Reconstitution of images measured with the Gaussian random matrix under compression ratio at (**a**) 0.1, (**b**) 0.2, (**c**) 0.3, (**d**) 0.4, (**e**) 0.5, and reconstitution of images measured with adaptive measurement matrix under compression ratio at (**f**) 0.1, (**g**) 0.2, (**h**) 0.3, (**i**) 0.4, (**j**) 0.5, the reconstruction algorithm is TVAL3 and the resolution of the image is 64×64 for all.

Figure 3a–e show the images of different compression ratios measured by the Gaussian random matrix. Figure 3f–j show the images of different compression ratios measured by the adaptive measurement matrix.

From Figure 3 we can conclude that, CS can recover images extremely well below the Nyquist sampling frequency. With the increasing of sampling rate, the quality of imaging measured with both matrices have improved greatly. At extremely low compression ratios, the image obtained by the Gaussian random matrix is fuzzy, but the image measured through the adaptive measurement matrix can still be reconstructed with a high quality.

**Figure 4:**
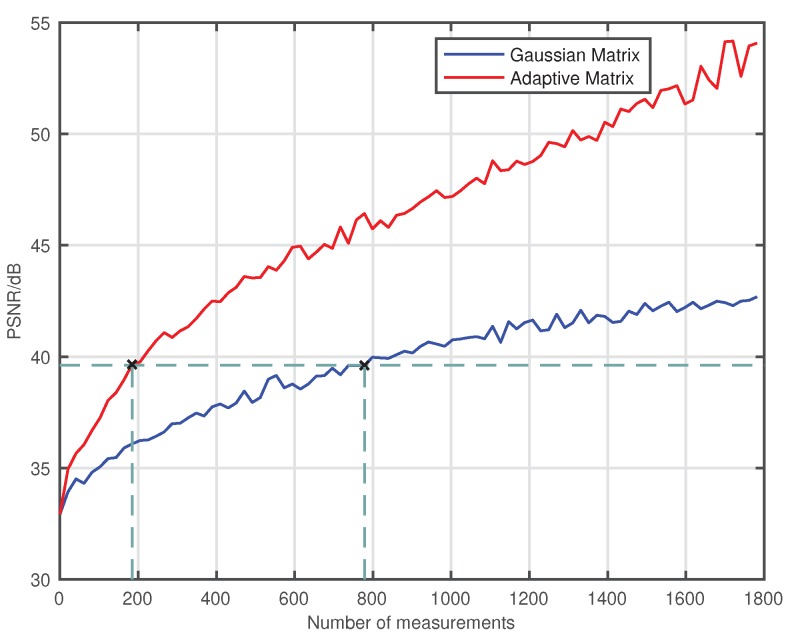
PSNR of image reconstructed with number of measurement time from 0 to 1800 with a step of 20, which measured with the Gaussian random matrix and the adaptive measurement matrix. When the PSNR of both image is 39.6 dB, the image measured with the Gaussian random matrix requires 779 measurements, while the image measured with the adaptive measurement matrix requires only 184 measurements.

Theoretically, the PSNR are often used for evaluating the similarity between two images. If the original image is used as one of the reference images, the PSNR can be used to evaluate the quality of the reconstructed image. However, the image of the object is on the DMD, we could not get the original image of the object. For approximation processing, we select a reconstructed image that is integrated over a long period of time, shown in Figure 3j, as a reference image, which is similar to the original image of the object. Then, we perform the image reconstruction with a number of measurements from 0 to 1800 with a step of 20. The results of the PSNR are given in Figure 4. With the development of the number of measurements and time, the PSNR of the image that is measured by both the adaptive measurement matrix and the image measured by the Gaussian random matrix increased accordingly. When the PSNR of the image measured by these two matrices is 39.6dB, the image measured with the Gaussian random matrix requires 779 measurements, while the image measured with the adaptive measurement matrix only requires 184 measurements, which saves 76.37% time. Thus, we conclude that using the adaptive measurement matrix constructed by this method can greatly reduce the times of measurements and shorten the imaging time.

### 4.2. Effect of Reconstruction Algorithm on Imaging Quality

**Figure 5:**
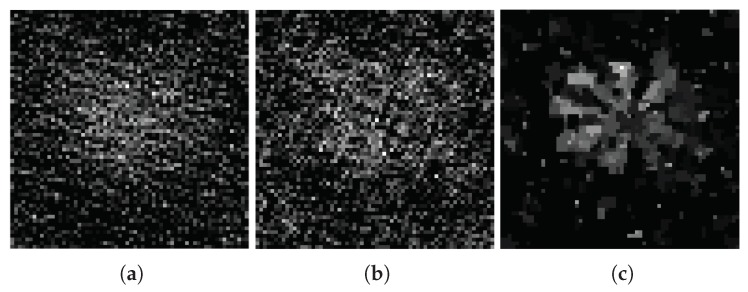
In the case of the sampling rate is 0.15, the image measured by Gaussian random matrix reconstructed by OMP (**a**), IHT (**b**), and TVAL3 (**c**) algorithms. The resolution of the image is all 64×64.

**Figure 6:**
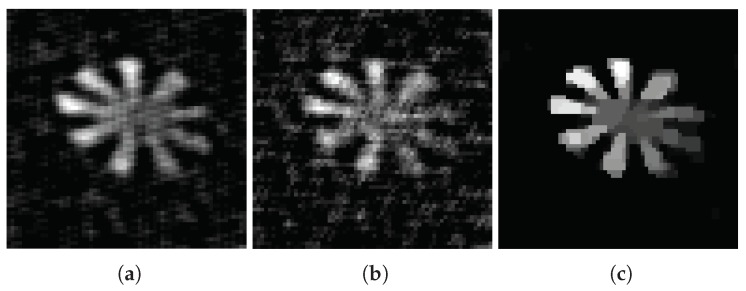
In the case of the sampling rate is 0.15, the image measured by adaptive measurement matrix reconstructed by OMP (**a**), IHT (**b**), and TVAL3 (**c**) algorithms. The resolution of the image is all 64×64.

**Figure 7:**
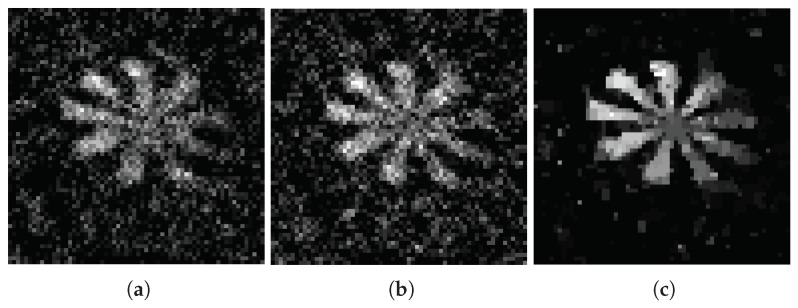
In the case of the sampling rate is 0.4, the image measured by Gaussian random matrix reconstructed by OMP (**a**), IHT (**b**), and TVAL3 (**c**) algorithms. The resolution of the image is all 64×64.

**Figure 8:**
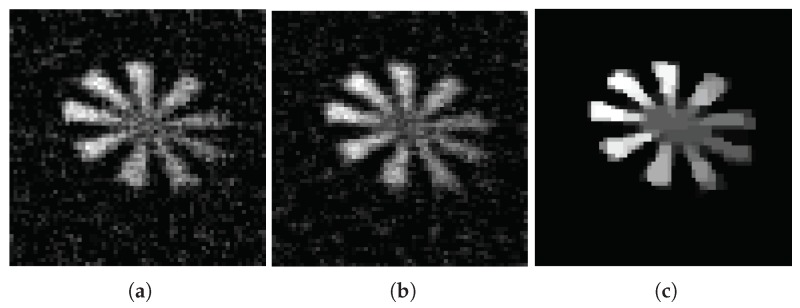
In the case of the sampling rate is 0.4, the image measured by adaptive measurement matrix reconstructed by OMP (**a**), IHT (**b**), and TVAL3 (**c**) algorithms. The resolution of the image is all 64×64.

The images reconstructed by OMP(a), IHT (b), and TVAL3 (c) algorithms are shown in Figure 5 (measured with the Gaussian random matrix) and Figure 6 (measured with the adaptive random matrix) under compression ratio of 0.15. When the compression ratio is 0.4, the images reconstructed by OMP (a), IHT (b), and TVAL3 (c) algorithms are shown in Figure 7 (measured with the Gaussian random matrix) and Figure 8 (measured with the adaptive measurement matrix).

Comparing Figure 5, Figure 6, Figure 7 and Figure 8, we can conclude that the recovery effect of TVAL3 is significantly better than that of the other two algorithms under the same conditions. Additionally, the reconstruction effect using the adaptive measurement matrix is obviously better than that of the Gaussian random matrix under the same sampling rate. From this, it can be concluded that imaging with the adaptive measurement matrix can perform high-quality measurements of the image at an extremely low sampling rate regardless of which reconstruction algorithm is used.

### 4.3. Anti-Noise Ability of Adaptive Measurement Matrix

**Figure 9:**
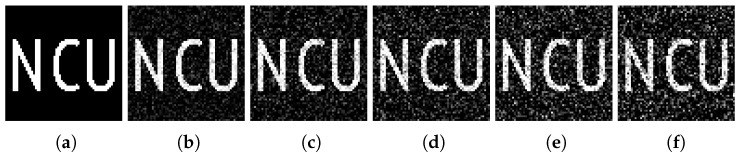
Image added Gaussian noise with a mean of 0 and variance of (**a**) 0, (**b**) 0.02, (**c**) 0.04, (**d**) 0.06, (**e**) 0.08, and (**f**) 0.1. The resolution of the images are all 64×64.

**Figure 10:**
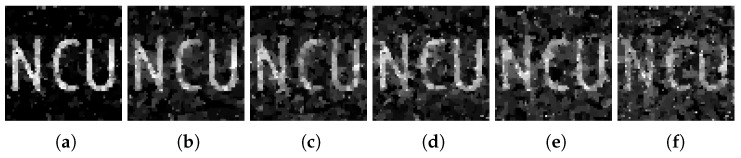
When the sampling rate is 0.1, the image reconstructed from the original image measured by Gaussian random matrix added with a mean of 0 and variance of (**a**) 0, (**b**) 0.02, (**c**) 0.04, (**d**) 0.06, (**e**) 0.08, and (**f**) 0.1. The reconstructed algorithm is TVAL3. The resolution of the image is 64×64 for all.

**Figure 11:**
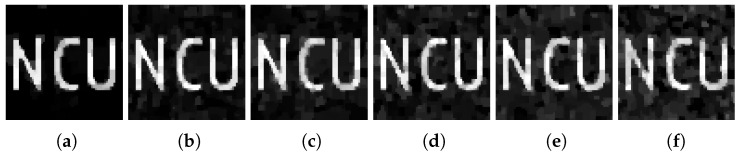
When the sampling rate is 0.1, the image reconstructed from the original image measured by adaptive measurement matrix added with a mean of 0 and variance of (**a**) 0, (**b**) 0.02, (**c**) 0.04, (**d**) 0.06, (**e**) 0.08, and (**f**) 0.1. The reconstructed algorithm is TVAL3. The resolution of the image is 64×64 for all.

The practical single-photon compressed imaging system often contains various types of noise, such as dark counts from PMT and counts from background light. In order to conduct a research of the anti-noise ability of the adaptive measurement matrix, we conducted a simulation experiment. First, we added Gaussian noises with a mean of 0 and variance of 0 to 0.1 into the original image as shown in Figure 9. Then, after conducting the CS process, the image measured by Gaussian random matrix and adaptive measurement matrix reconstructed by TVAL3 algorithms is shown in Figure 10 and Figure 11, respectively.

**Figure 12:**
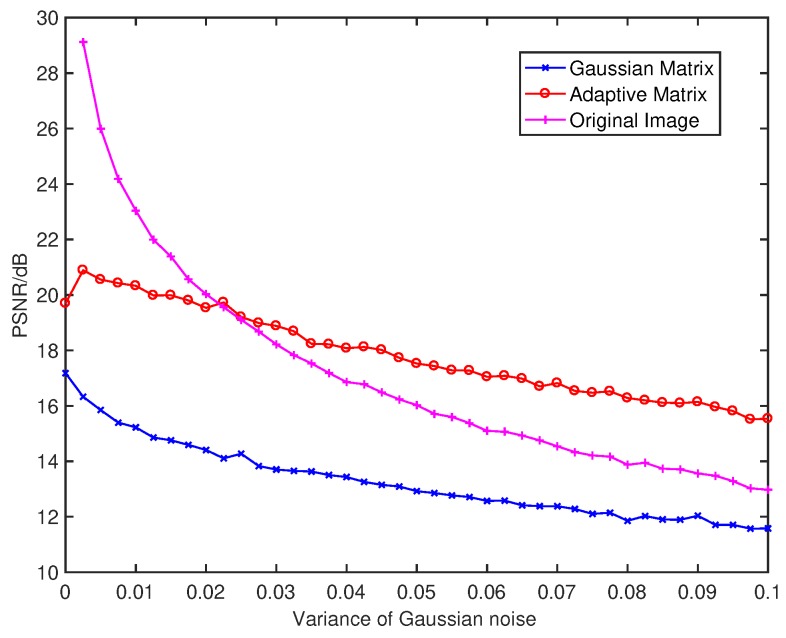
PSNR of image reconstructed from the original image added with Gaussian noise, which with a mean of 0 and different variance of 0.0–0.1 by using the adaptive matrix measure with the Gaussian random matrix and the adaptive matrix, the resolution of the image is all 64×64, and the reconstructed algorithm is TVAL3.

Compare with Figure 10 and Figure 11, the image measured by the adaptive measurement matrix have more distinguishable details. The PSNR of each image is shown in Figure 12, from which we can conclude that the image measured by our adaptive measurement matrix is more precise at any signal to noise ratio. Even when the variance of the noise is large enough, the quality of image measured by our adaptive measurement matrix is obviously better than that of the noise-added image. The change of PSNR of the image acquired by the adaptive measurement matrix is smaller with the change of variance of the Gaussian noise, which proved that the adaptive measurement matrix has a better anti-noise performance.

## 5. Conclusions

A single-photon compressed imaging system based on single photon counting technology and CS is built. We proposed a fast and effective adaptive method for that system. First, we reconstructed the rough image obtained previously in sparse basis as a priori information. Then, we set up a special and smart threshold matrix using large sparse coefficients of the image in sparse basis. The adaptive measurement matrix is obtained by modifying the original Gaussian random matrix with the specially designed threshold matrix. In this process, only one level of sparse representation is needed to set up the adaptive measurement matrix. Thus, less computations and adaptations are needed and the process can be achieved quickly. In addition, there is no decline in the resolution of any space area, which allows the whole space area to be be reconstructed with in extremely high quality. The experimental results show that under different measurement times and different reconstruction algorithms, the reconstruction effect of the image measured using the adaptive measurement matrix is obviously better than that of the Gaussian random matrix. The simulation shows that the adaptive measurement matrix has a better anti-noise performance.
